# Gluten-free is not enough

**DOI:** 10.31744/einstein_journal/2026CE2428

**Published:** 2026-05-21

**Authors:** Alexandre Rodrigues Marra

**Affiliations:** 1 Hospital Israelita Albert Einstein São Paulo SP Brazil Hospital Israelita Albert Einstein, São Paulo, SP, Brazil.; 2 University of Iowa Health Care Iowa City IA United States University of Iowa Health Care, Iowa City, IA, United States.

Dear Editor,

For the past ten years, my family and I have lived entirely gluten free. It is entirely possible to live without gluten with no nutritional compromise. The real challenges lie not in nutritional adequacy, but in safety, diagnosis, and social inclusion.

My youngest son was nine years old at the time he was diagnosed with celiac disease. For about a year and a half before that, he had been followed by a dermatologist for what was believed to be atopic dermatitis, primarily affecting the palms of his hands and the soles of his feet. He had a known atopic background. His bowel habits were never strikingly abnormal, consisting of one bowel movement per day, usually soft but not frankly diarrheal.

Three months before the diagnosis, however, something changed. He developed profound fatigue while playing soccer and began to fall ill repeatedly, with short episodes of fever, diarrhea, and vomiting lasting less than a week, followed by brief improvement and subsequent relapse. The diagnostic suspicion was not straightforward, largely because throughout this entire period, during both improvement and deterioration, he continued to be exposed to gluten daily.

Celiac disease is a genetic, autoimmune condition in which disease activity does not occur without exposure to gluten, whether derived from wheat, barley, or rye. There is no pharmacological treatment. The only effective therapy is strict, lifelong avoidance of gluten, relying exclusively on foods that are truly gluten free.

## This is where danger lies for the unwary

Naturally gluten-free foods are rarely the problem. Processed foods labeled as gluten-free often are. Regulatory definitions allow foods labeled gluten-free to contain up to 20 parts per million of gluten.^(1)^ Some countries adopt stricter limits, below 10 parts per million.^(2)^ For patients with celiac disease, these differences matter.

Since my son's diagnosis, I have learned to read every label, scrutinize every ingredient list, and carefully check for gluten-free claims. In the United States, manufacturers are not universally required to indicate whether a product is gluten free. Even reading ingredient lists is insufficient, because foods processed on shared equipment, particularly equipment used for wheat-containing products, can become contaminated despite containing no gluten-based ingredients.

Even when a label states that a product may contain traces of gluten, that product can no longer be considered gluten free. Traces may imply levels that exceed 20 parts per million. The same lack of transparency applies to pharmaceuticals, vitamins, and supplements, which may contain gluten-derived excipients without disclosure, as well as to cosmetics.^(3,4)^ Lipstick may contain gluten, a detail my son, now 19 years old, may eventually have to consider in intimate human relationships. These are not trivial concerns. They are part of daily life with celiac disease.

Diagnosis itself remains a major challenge. Screening relies on indirect methods, such as antibodies against gliadin and the more specific tissue transglutaminase antibodies of the IgA and IgG classes. In patients with IgA deficiency, negative serology does not exclude the disease. That was not the case for my son. All antibodies’ tests were positive, leading to confirmatory testing with duodenal biopsy.

Even then, another barrier emerged. His upper endoscopy appeared macroscopically normal. Only histopathologic examination of the biopsied mucosa revealed severe villous atrophy with complete loss of intestinal villi, corresponding to Marsh stage IV. Had biopsies not been performed because the mucosa appeared normal, his diagnosis could have been missed, exposing him to years of ongoing intestinal injury and long-term complications.

Celiac disease affects approximately 1% of the global population, although it remains underdiagnosed.^(1)^ In addition, there is a growing group of individuals with non-celiac gluten sensitivity, who test negative for celiac antibodies but experience symptoms related to gluten ingestion. There is no single diagnostic pattern that fits all cases. Celiac disease can be diagnosed at any age, from early childhood, when incidence peaks at around two years of age, to late adulthood.

Classically, celiac disease is associated with chronic diarrhea, but this occurs in only about half of patients. Because it is a systemic disease, it may also be present as severe anemia, unexplained fractures, or premature osteoporosis. Women younger than 40 years of age with osteoporosis should be evaluated for celiac disease. Fatigue, headaches, mood changes, irritability, and shortness of breath are common prediagnostic complaints. The most characteristic cutaneous manifestation is dermatitis herpetiformis, which can affect any area of the body. My son's dermatitis, however, was atopic rather than herpetiform.

Human beings are inherently social creatures. Studies consistently show that people who socialize more tend to report greater happiness.^(5)^ The COVID-19 pandemic made this painfully clear, as older adults and patients with Alzheimer's disease experienced measurable cognitive decline during periods of social isolation. Social gatherings, including tailgates and holidays such as Thanksgiving and Christmas, invariably revolve around food. Food is central to human connection, shared traditions, and cultural identity. Wheat, in particular, plays a central role in many diets.

Restaurants and private homes rarely have kitchens dedicated exclusively to gluten-free food. This is where risk becomes unavoidable. How can I safely offer my son a meal when I cannot be certain that gluten is absent?

Cross-contamination occurs at the level of parts per million. A gluten-free meal must remain below 20 parts per million even after preparation. In practice, this is nearly impossible to verify. Home testing devices exist, but they have significant limitations in sensitivity and reliability. Some have already been discontinued. None can replace laboratory-based testing or robust regulatory labeling.

When a person with celiac disease inadvertently ingests gluten, the reaction is neither immediate nor allergic in nature. This is not a gluten allergy. Symptoms often appear 12 to 24 hours later, sometimes after several subsequent meals, making it difficult to identify the offending food. Rapid finger-prick tests exist to detect celiac-related antibodies, such as anti- tissue transglutaminase antibodies, but these tests are useful only for screening. They do not confirm the diagnosis and are entirely unhelpful after acute gluten exposure. Antibodies reflect chronic, sustained immune activation, not isolated contamination.

The limitation imposed by celiac disease is therefore not only dietary, but also social. For true safety, the best option is often to host friends at home and eat exclusively in one's own kitchen. When eating outside the home, the safest approach may be to bring one's own food, prepared in a strictly gluten-free environment. Patients should not feel intimidated by social pressure. There is no such thing as eating just a little. There is no gradual desensitization. There is no pill or enzyme equivalent to lactase for lactose intolerance. Although many patients with celiac disease also develop lactose intolerance, that is not the central problem.

What needs to change is not the patient, but the environment. For the substantial portion of the population that cannot consume gluten safely, we need more care, more awareness, and more truly safe options.

We need more safe gluten-free food. However, gluten-free alone is not enough.

## Data Availability

The underlying content is contained within the manuscript.
